# Benefits of probabilistic sensitivity analysis – a review of NICE decisions

**DOI:** 10.3402/jmahp.v1i0.21240

**Published:** 2013-08-06

**Authors:** Erpur Adalsteinsson, Mondher Toumi

**Affiliations:** 1 Novo Nordisk, Søborg, Denmark; 2 Department of Decision Science, University Claude Bernard Lyon, Lyon Cedex, France

**Keywords:** probabilistic sensitivity analysis, NICE, health technology assessment, decision

## Abstract

**Objective:**

Since 2004, the National Institute of Health and Clinical Excellence (NICE) has required manufacturers to conduct a probabilistic sensitivity analysis (PSA) in their technology appraisals. The objective of this review is to assess the cost-effectiveness of different technology appraisals and compare them with the actual decision made by the NICE based on PSA.

**Methods:**

The search term ‘probabilistic sensitivity analysis’ was used on the NICE home page (25 January 2012). The appraisals identified in the search were assessed and subjected to further review, if a probability of being cost-effective was provided, regardless of the threshold indicated. If several probabilities were provided, the number provided by the evidence review group was used. If several scenarios were presented, the base case scenario was chosen. Finally, the probabilities of being cost-effective were compared with the actual decision made, which could result in two outcomes: recommended or not recommended.

**Results:**

A total of 31 assessments were included for the final review. The results were plotted on a graph to illustrate whether there was a relationship between the PSA outcomes and the final recommendation. The assessments were ranked according to their probability of being cost-effective.

**Conclusion:**

A higher probability of a technology being cost-effective was correlated with more positive decision-making. There appeared to be a clear threshold at which technologies with a 40% certainty of being cost-effective tended to be recommended, whereas those below the threshold were not recommended. The reports suggested that the incremental cost-effectiveness ratios (ICER) estimate was not a robust driver of decision-making. A NICE applicant should pay increased attention to the PSA in addition to the ICER estimate.

Health technology assessment and economic evaluation are increasingly being used to inform decision-making and allocate limited funding for health care. These methods are being increasingly implemented as health care costs are on the rise in an ageing population. Most, if not all, decision-makers are uncertain while evaluating new health care technologies. This uncertainty is due to several factors, including a lack of long-term data on efficacy and safety, and a lack of credible data on costs and utilities. Economic modelling is inevitable in bridging the gap between clinical trial settings, often with surrogate endpoints, and the decision-makers’ need for making decisions on how the technology is likely to work in real-life settings (1).

Economic modelling often results in incremental cost-effectiveness ratios (ICER) compared to other interventions. However, this value is linked to uncertainties due to several factors, such as variability in the underlying data, choice of economic model and validity of the results for the intended population. A sensitivity analysis is a tool that aims to address this uncertainty. There are numerous types of sensitivity analyses. The deterministic sensitivity analysis, involves varying one (univariate) or more (multivariate) variables simultaneously and plotting the results depending on various scenarios.

However, the use of this type of sensitivity analysis is limited, as it does not reveal the likelihood of the occurrence of each scenario. To demonstrate the possibility of a technology being cost-effective at a certain threshold, a probabilistic sensitivity analysis (PSA) is required in which distributions of the variables being modelled can be set, although PSA as such is also useful for testing the robustness of the models. This analysis aims to demonstrate the robustness of the ICER with regard to input. The results can be expressed as the likelihood of being cost-effective at a certain threshold. However, a PSA does not have the ability to reduce uncertainty regarding the analytical method being used (2).

Since 2004, the National Institute of Health and Clinical Excellence (NICE) has required manufacturers to conduct a PSA in their technology appraisals. The NICE is, among other things, responsible for issuing clinical guidelines and conducting single technology appraisals in England and Wales and is one of the most prominent Health Technology Assessment agencies in the world. The results of a PSA are typically expressed as a cost-effectiveness acceptability curve (3). Manufacturers should clearly justify why certain variables in the analysis were included or excluded.

## How is PSA used by the NICE?

**Fig. 1:**
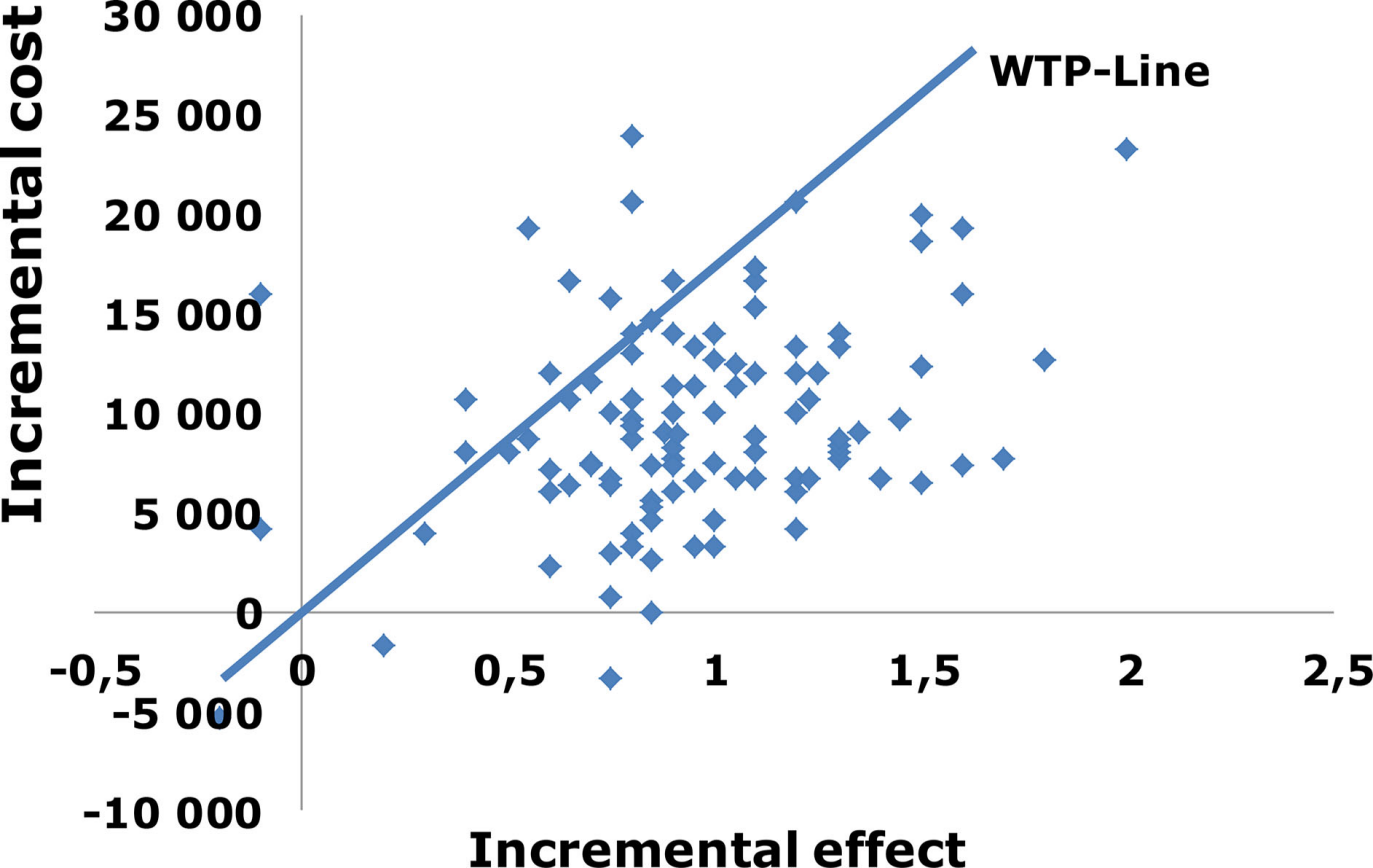
PSA on a cost-effectiveness plane with the diagonal willingness to pay line.

As discussed, PSA has the advantage of indicating the probability of a technology being cost-effective at various thresholds. A high probability of being cost-effective should lead to a more positive outcome in a technology appraisal, whereas the opposite should apply for a low probability. As decision-makers are likely to be risk-averse, the actual distribution of the results should also play an important role. Figure 1 shows the results of a PSA, which are typically presented in a cost-effectiveness plane with a willingness to pay (WTP) curve that passes through the origin. This curve is often referred to as the acceptability curve. The higher the WTP, the steeper the acceptability curve. The results are usually concentrated in the upper right corner when a new technology is better and also when it is costlier than an older technology. There are also methodological challenges with results outside of the upper right corner. Interventions in the lower left quadrant are less effective but have lower costs, which mean that a high ICER is preferred over a low ICER. If a new technology is introduced that is less effective than the previous technology, it should come with as large an increase in savings as possible. ICER in the other two quadrants can hardly be graded because they are either good (i.e. favourable effect and negative cost) or bad (i.e. favourable cost and negative effect). Therefore, results concentrated around one point estimate in the upper right corner should provide the least uncertainty concerning the ICER estimate (4).

The objective of this review is to assess the outcomes of different probabilities of being cost-effective and to compare them with the actual decision made by the NICE regardless of what cost-effectiveness thresholds are applied. The institute typically accepts technologies with an ICER ranging between £20,000 and £30,000 per Quality Adjusted Life Year (QALY), although higher values may apply for end-of-life treatments (5). It is currently poorly understood as to how the results of PSA are used in practice by the NICE.

The slope of the WTP line determines the threshold.

## Method

The search term ‘probabilistic sensitivity analysis’ was used on the NICE home page (25 January 2012) and resulted in 90 items. In total, the NICE has published more than 250 technology appraisals. No additional search was deemed to be necessary as all technology appraisals were published on the NICE home page. The results found in the search were assessed and included for further review, if a probability of being cost-effective was provided, regardless of the threshold indicated. If several probabilities were provided, the number provided by the evidence review group was used rather than the number provided by the manufacturer, because this number is more likely to be used in decision-making. If several scenarios were presented, the base case scenario was chosen. All of the mentioned thresholds were presented, as NICE appeared to have used several thresholds, often in the same reports. Finally, the probabilities of being cost-effective were compared with the actual decision made, which could result in two outcomes: recommended or not recommended.

## Results

**Fig. 2:**
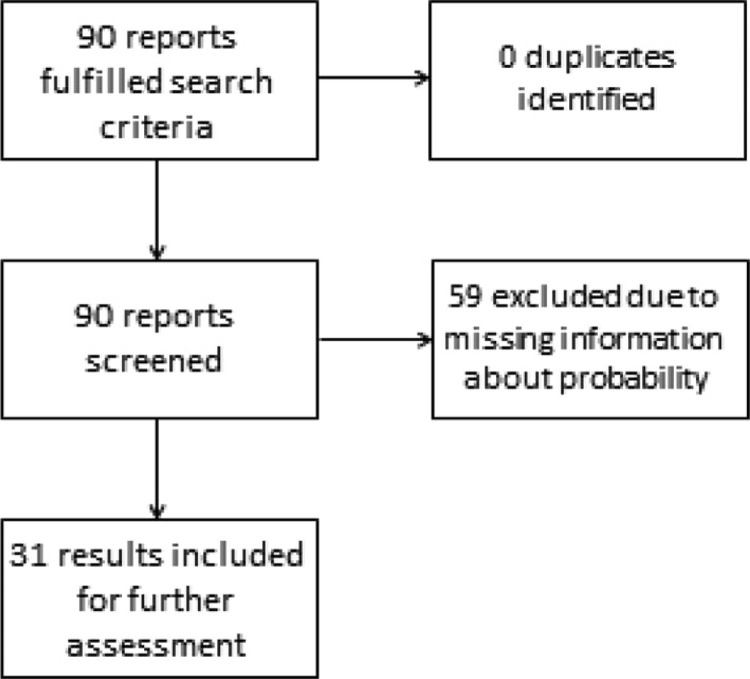
Flow chart of the included health technology assessments.

Figure 2 illustrates the search, which led to 31 results being included for the final review. Most of the initial search results had to be excluded due to lack of information about probability of cost-effectiveness.

Approximately two-thirds of the assessments contained no details about the likelihood of cost-effectiveness.

The 31 assessments came from a total of 30 reports, as one report (TA 118) contained probabilities from more than one technology. The reports were on various technologies, with cancer treatments constituting the dominant share (16 of the 31 decisions), followed by 5 decisions on inflammatory diseases. Table I illustrates the results.

**Table I:** Summary of the findings

Technology	Disease	Year	TA Nr	Threshold in £ (probability of being cost-effective)	Recommended
Bevacizumab	Colorectal cancer	2007	118	30,000 (0%)	No
Omalizumab	Severe asthma	2010	201	20,000 (0%), 30,000 (0%)	No
Fulvestrant	Breast cancer	2011	239	20,000 (2%)	No
Cetuximab	Colorectal cancer	2007	118	30,000 (10%)	No
Adalimumab	Psoriasis	2008	146	30,000 (16%)	No
Pegaptanib	Macular degeneration	2008	155	20,000 (17%), 30,000 (58%)	No
Somatropin	Growth failure	2010	188	20,000 (22%), 30,000 (95%), 5,000 (100%)	Yes
Trabectedin	Ovarian cancer	2011	222	30,000 (23%), 70,000 (50%)	No
Ofatumumab	Leukaemia	2010	202	30,000 (28%)	No
Infliximab	Psoriasis	2008	134	30,000 (38%)	Yes
Imatinib	Gastric cancer	2010	196	20,000 (41%), 30,000 (74%)	No
Sunitinib	Gastric cancer	2009	179	30,000 (42%)	Yes
Entecavir	Hepatitis B	2008	153	20,000 (45%)	Yes
Certolizumab	Rheumatoid arthritis	2010	186	20,000 (48.7%)	Yes
Ranibizumab	Macular oedema	2011	237	20,000 (49.3%), 30,000 (76.8%)	No
Topotecan	Cervical cancer	2009	183	20,000 (50%), 30,000 (88%)	Yes
Sunitinib	Renal cancer	2009	169	30,000 (51%)	Yes
Everolimus	Renal cancer	2011	219	50,000 (52.7%)	No
Prucalopride	Chronic constipation	2010	211	20,000 (55%), 30,000 (60%)	Yes
Pemetrexed	Lung cancer	2010	190	50,000 (57.7%)	Yes
Tenofovir	Hepatitis B	2009	173	20,000 (58%)	Yes
Erlotinib	Lung cancer	2008	162	30,000 (68%)	Yes
CRT-P	Heart failure	2007	120	20,000 (72%), 30,000 (95%)	Yes
Ranibizumab	Macular degeneration	2008	155	20,000 (72%), 30,000 (97%)	Yes
Rituximab	Leukaemia	2010	193	20,000 (75%), 30,000 (94%)	Yes
Dasatinib	Leukaemia	2012	241	30,000 (81%)	No
Rituximab	Lymphoma	2011	226	20,000 (84%), 30,000 (99.7%)	Yes
Etanercept	Ankylosing spondylitis	2008	143	15,000 (88%),	Yes
Peginterferon	Hepatitis C	2010	200	20,000 (90%), 30,000 (90%)	Yes
Rituximab	Lymphoma	2003	65	23,400 (95%)	Yes
Aripiprazole	Schizophrenia	2011	213	20,000 (96%)	Yes

TA Nr is the technology appraisal number. The probability of being cost-effective is ranked in order, starting from the lowest probability. In some cases, several thresholds and probabilities are presented.

**Fig. 3:**
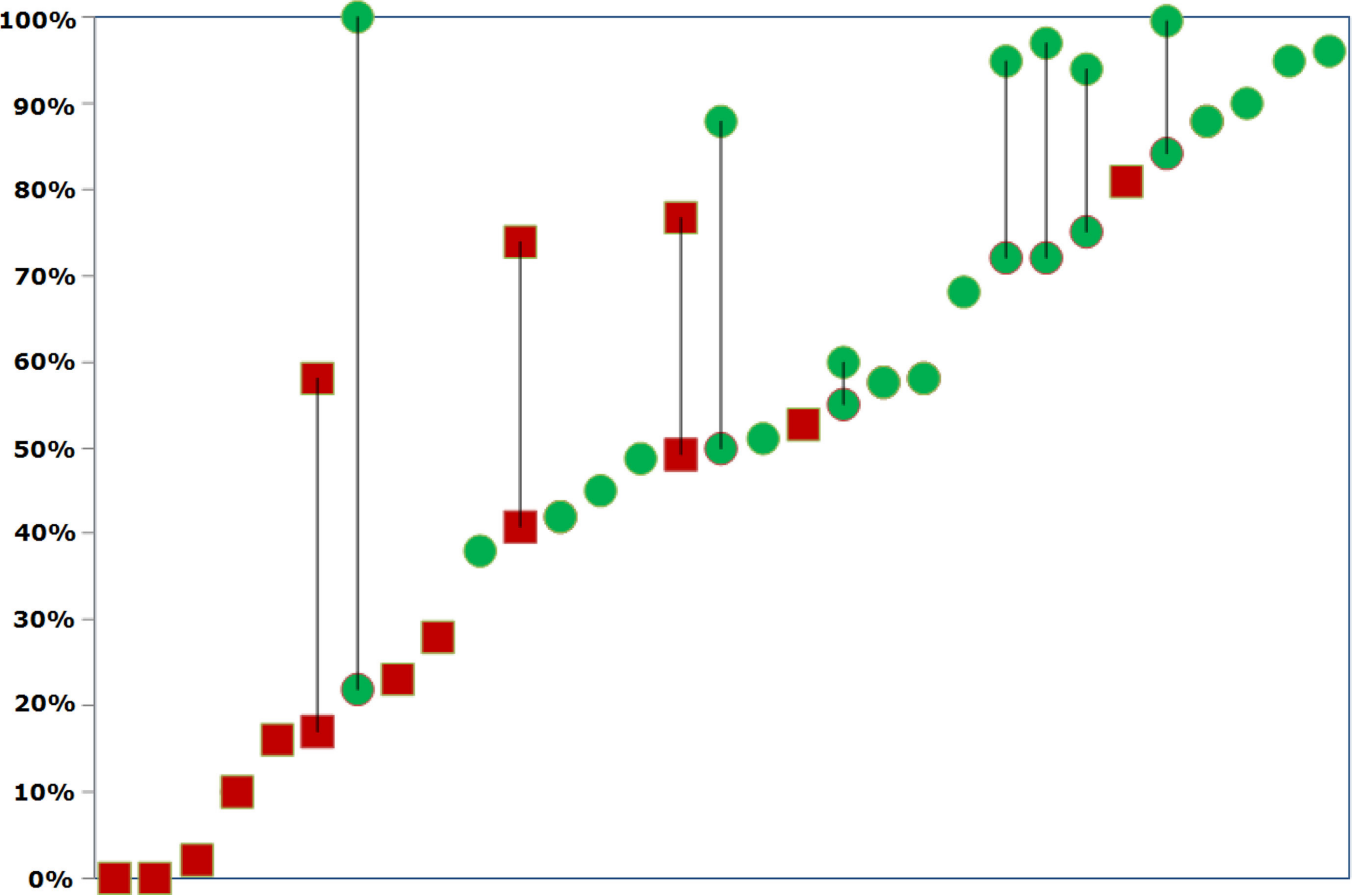
Probabilities ranked in order and graphically demonstrated.

As shown, a variety of thresholds were indicated in the reports, ranging from £15,000 to £70,000, and in some reports, several thresholds were mentioned. The most common thresholds were £20,000 and/or £30,000. The results were plotted to illustrate whether there was a relationship between the PSA outcomes and the final recommendation. The assessments were ranked according to their probability of being cost-effective. If several thresholds were mentioned, the lowest threshold was chosen to rank them, but both the lowest and the highest thresholds are illustrated in Fig. 3. In this figure, dot represents recommended and square represents not recommended. The vertical line between the dots indicates that several thresholds and probabilities were presented.

**Fig. 4:**
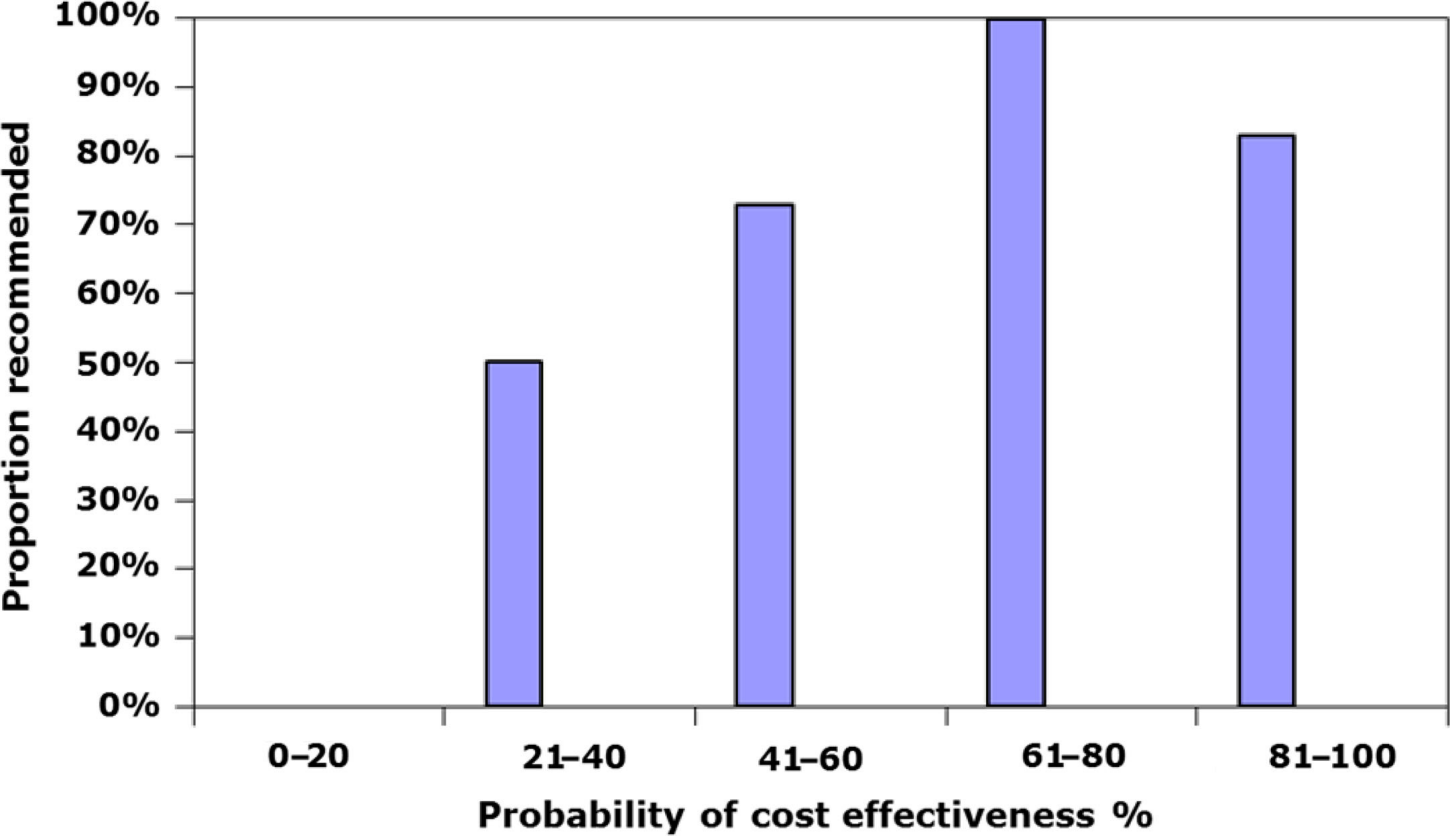
The proportion recommended versus the probability of cost-effectiveness.

As shown, there seemed to be only a few exceptions to the pattern that a high probability of cost-effectiveness at the mentioned thresholds led to a positive outcome, whereas a low probability led to a negative recommendation. There appeared to be a threshold at which the decision-maker wanted a probability of 40% or more to feel safe about the likelihood of cost-effectiveness. The one exception in which a low probability was recommended was growth hormone, for which several thresholds were mentioned, and for the highest threshold of £50,000, the probability of being cost-effective was found to be 100%. Infliximab was also recommended with a 38% probability of being cost-effective. Four technologies did not obtain a positive outcome, even though a high probability was presented. For imatinib, the health economic evidence was found to be uncertain; for ranibizumab, the committee found uncertainties in the economic model; and for both dasatinib and everolimus, it was stated that there were uncertainties in the ICER calculation. Figure 4 illustrates the relationship between the likelihood of cost-effectiveness and the proportion of recommendations.

None of the treatments were recommended when the probability was below 20%.

## Discussion

A PSA is more of an informative statistical analysis than simpler versions of statistical analysis, such as the deterministic method. The method not only addresses the confidence interval of the point estimate of an ICER but also, in combination with an acceptability curve, provides the decision-maker with further confidence that the results are robust. The main argument is that a PSA is the only sensitivity analysis that can potentially address uncertainties in all inputs, not merely confined to a subset (6).

For advanced economic models, for example, Markov models with numerous transitions, performing the required PSA can be time-consuming. It can also require extensive computer resources, as in some cases the submitting company does not own the model being used, making it more complicated to perform.

It should therefore be fair to assume that the requirement to perform a PSA gives the decision-maker increased confidence compared with a simple point estimate of an ICER. In this review, no comment was found in the reports about the distribution of the PSA. If a deterministic analysis gives an ICER below a certain threshold, there is, on average, at least a 50% probability that the technology will be cost-effective. These results show that a 40% probability of being cost-effective is enough in most cases, with a few exceptions. The three results that were accepted between 40% and 50% probability are also worth noting. The technologies accepted were against gastric cancer, rheumatoid arthritis and hepatitis B, which are considered severe conditions. This shows that the NICE was willing to accept a point estimate slightly above the normal range for severe conditions with few effective treatment options. In the reports, it was surprisingly not argued as to why different values were used as basis for probability calculations. The only disease area where there generally is a higher WTP is for end-of-life treatments (7).

A limitation of this analysis is that only a fraction of the published technology reports could be included due lack of information about probability of cost-effectiveness. The conclusion has also not been tested in a statistical model, for example, regression analysis of potential confounders. One could also argue that the deterministic cost per QALY is likely to be highly correlated with the probability of being cost-effective at a given threshold and therefore is also important for decision-making. The included assessments were considered too few to perform an analysis of subgroups such as which committee made the decision, which group produced the report, and so on.

In this review, the reports from the evidence review groups were not assessed; the arguments from the committee were the main focus used to assess the committee evaluation of the PSA. Decision-making for recommending a drug at NICE is multifactorial, and some criteria could be implicit, while here we only focused on the PSA. However, it is clear that the PSA is a critical factor for decision-makers. Similar research has revealed similar results, in which the distribution of the ICER is hardly commented on in the assessments (8).

## Conclusion

A PSA has been mandatory in technology appraisals since 2004 for the NICE. A higher probability of a technology being cost-effective seems to logically correlate with more positive decision-making, and there appears to be a threshold at which technologies with a 40% certainty of being cost-effective tend to be recommended, whereas those below this threshold are not recommended. Although the distribution of the PSA was rarely commented on, it is a critical issue for decision-makers. A NICE applicant should pay increased attention to the PSA in addition to the ICER estimate.

## Contributor statement

Erpur Adalsteinsson contributed with conception, design, search, analysis of data, drafting of article and approval of final manuscript.

Mondher Toumi contributed with design, analysis of data, critical proofreading and approval of final manuscript.

## Article summary

### Article focus

- How is probabilistic sensitivity analysis used in decision-making by the NICE?

## Key messages

- NICE applicants should pay attention to probabilistic sensitivity analysis as this was seen to be a key driver of decision-making.

## Strengths and limitations

- Systematic search of all published technology appraisals performed by NICE.
- Only 31 technology appraisals could be included in the final review.

## Data sharing statement

There is no additional data available.

## Disclaimer

Statements in this article are solely the opinions of the authors and not of their employers.

Erpur Adalsteinsson has completed the Unified Competing Interest form at www.icmje.org/coi_disclosure.pdf (available on request from the corresponding author) and declares that E. A. has relationships with Novo Nordisk that might have an interest in the submitted work in the previous 3 years. Mondher Toumi consulting for various pharmaceutical companies and authorities. Their spouses, partners or children have no financial relationships that may be relevant to the submitted work; and E. A. an M. T. have no non-financial interests that may be relevant to the submitted work.
